# An Exon-Specific Small Nuclear U1 RNA (ExSpeU1) Improves Hepatic OTC Expression in a Splicing-Defective *spf*/*ash* Mouse Model of Ornithine Transcarbamylase Deficiency

**DOI:** 10.3390/ijms21228735

**Published:** 2020-11-19

**Authors:** Dario Balestra, Mattia Ferrarese, Silvia Lombardi, Nicole Ziliotto, Alessio Branchini, Naomi Petersen, Piter Bosma, Mirko Pinotti, Stan F. J. van de Graaf

**Affiliations:** 1 Department of Life Sciences and Biotechnology and LTTA, University of Ferrara, 44121 Ferrara, Italy; frrmtt1@unife.it (M.F.); lmbslv@unife.it (S.L.); or nicole.ziliotto@unimib.it (N.Z.); brnlss@unife.it (A.B.); pnm@unife.it (M.P.); 2 School of Medicine and Surgery, University of Milano-Bicocca, 20900 Milan, Italy; 3 Tytgat Institute for Liver and Intestinal Research and Department of Gastroenterology and Hepatology, Amsterdam UMC, University of Amsterdam, 1105 AZ Amsterdam, The Netherlands; n.petersen@amc.uva.nl (N.P.); p.j.bosma@amsterdamumc.nl (P.B.); k.f.vandegraaf@amsterdamumc.nl (S.F.J.v.d.G.); 4 Amsterdam Gastroenterology Endocrinology Metabolism, Amsterdam UMC, 1105 AZ Amsterdam, The Netherlands

**Keywords:** ornithine transcarbamylase deficiency, OTCD, splicing, U1, mice, AAV

## Abstract

*OTC* splicing mutations are generally associated with the severest and early disease onset of ornithine transcarbamylase deficiency (OTCD), the most common urea cycle disorder. Noticeably, splicing defects can be rescued by spliceosomal U1snRNA variants, which showed their efficacy in cellular and animal models. Here, we challenged an U1snRNA variant in the OTCD mouse model (*spf*/*ash*) carrying the mutation c.386G > A (p.R129H), also reported in OTCD patients. It is known that the R129H change does not impair protein function but affects pre-mRNA splicing since it is located within the 5′ splice site. Through in vitro studies, we identified an Exon Specific U1snRNA (ExSpeU1^O3^) that targets an intronic region downstream of the defective exon 4 and rescues exon inclusion. The adeno-associated virus (AAV8)-mediated delivery of the ExSpeU1^O3^ to mouse hepatocytes, although in the presence of a modest transduction efficiency, led to increased levels of correct OTC transcripts (from 6.1 ± 1.4% to 17.2 ± 4.5%, *p* = 0.0033). Consistently, this resulted in increased liver expression of OTC protein, as demonstrated by Western blotting (~3 fold increase) and immunostaining. Altogether data provide the early proof-of-principle of the efficacy of ExSpeU1 in the *spf*/*ash* mouse model and encourage further studies to assess the potential of RNA therapeutics for OTCD caused by aberrant splicing.

## 1. Introduction

Ornithine transcarbamylase deficiency (OTCD, OMIM #311250), the most common urea cycle disorder with an estimated incidence of 1:40,000–70,000, is caused by mutations in the X-linked *OTC* gene encoding the mitochondrial matrix homo-trimeric enzyme that catalyzes the synthesis of citrulline, an essential step to convert the neurotoxic ammonia into urea. Therefore, OTCD is associated with hyperammonemia leading to encephalopathy in the first days of life and, if untreated, coma and death [1]. Besides liver transplantation [2], there is no cure for OTCD but only treatments to limit hyperammonemia (low-protein diet, nitrogen scavengers, L-Arginine supplementation) and hemodialysis in extreme states [3], with a five-year survival of ~25% [4]. Several efforts have been made to develop an effective therapy, including gene therapy [5] and genome editing [6], but the objective has not been reached yet.

Among the *OTC* nucleotide changes reported in OTCD patients, those predicted to affect pre-mRNA splicing are generally associated with the most severe and early (neonatal) onset of the disease [7,8] and represent preferred candidates for RNA therapeutics [9]. Among these approaches, variants of the U1snRNA, the RNA components of the spliceosomal ribonucleoprotein U1RNP that drives 5′ splice site (5′ss) recognition, have been shown to rescue exon-skipping mutations in several cellular models of human disease [10,11,12,13,14], and also in mouse models [15,16]. Importantly, different studies demonstrated that unique U1snRNAs targeting intronic sequences downstream of a defective exon (Exon specific U1snRNA, ExSpeU1) can rescue several exon-skipping mutations at 5′ss, 3′ss or within the exon, and this was proven both in vitro and in vivo [17,18,19,20,21]. Very recently, we have provided the early proof of principle of efficacy in vivo of U1snRNA variants for another metabolic disorder, hereditary tyrosinemia type I [22]. In this animal model, the delivery of a compensatory U1snRNA partially rescued the causative splicing mutation at both RNA and protein levels and resulted in a slightly prolonged mice survival.

Altogether this knowledge prompted us to challenge the U1snRNA-based correction strategy in the OTCD mouse model (*spf*/*ash*) [23,24] that carries the c.386G > A (p.R129H) mutation in the X-linked *OTC* gene. The predicted p.R129H amino acid substitution has no impact on mitochondrial OTC import, subunit assembly, or enzyme activity [24], whereas the corresponding c.386G > A change, occurring at the last nucleotide of *OTC* exon 4, and thus at the exon-intron junction, impairs OTC pre-mRNA splicing [23]. In particular, it remarkably decreases the proportion of correct transcripts and leads to the usage of a cryptic intronic 5′ss at position +49 or skipping of exon 4. Interestingly, this mutation has also been reported in OTCD patients, but in the slightly different human context, the change leads, besides exon 4 skipping, to the usage of cryptic 5′ss at position +5.

Here, by exploiting minigenes, we identified an ExSpeU1snRNA active in the mouse OTC context. Delivery of this ExSpeU1snRNA to hepatocytes, via an adeno-associated virus, partially rescued OTC splicing and protein expression in *spf*/*ash* mice, thus providing the early in vivo proof-of-principle of the efficacy of ExSpeU1 for OTCD caused by aberrant splicing. 

## 2. Results and Discussion

### 2.1. Identification of Active U1snRNA Variants by Minigene Assays 

**Figure 1:**
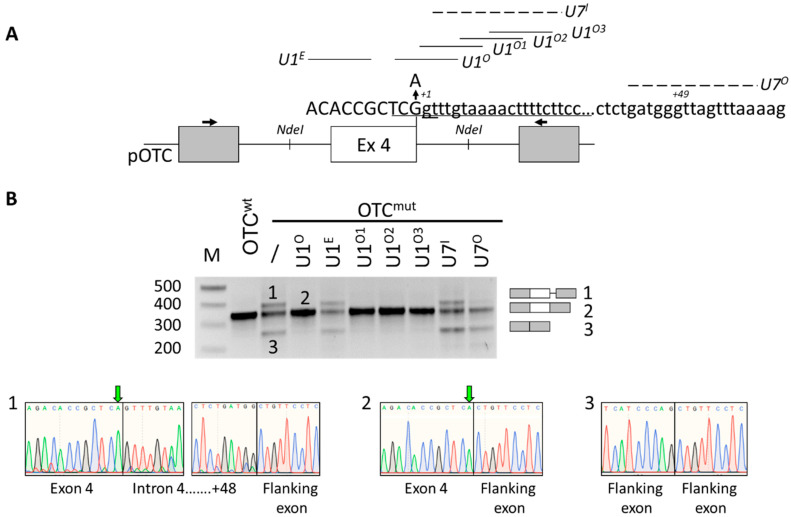
The *spf*/*ash* ornithine transcarbamylase (OTC) mutation can be efficiently rescued by U1snRNA variants. (**A**) Schematic representation of the mouse *OTC* genomic sequence cloned as minigene in the pTB vector. Exonic and intronic sequences are represented by boxes and lines, respectively. The sequences, with exonic and intronic nucleotides in upper and lower cases, respectively, report (i) the authentic 5′ss (position +1 within intron), and (ii) the intronic cryptic 5′ss (positions +49). The nucleotide change (G > A) leading to the spf/ash phenotype is indicated. The schematic representation of engineered U1 and U7 snRNAs, with relative binding sites, is reported. Primers used for RT-PCR are indicated by arrows. (**B**) Evaluation of mouse OTC alternative splicing patterns in Hepa1-6 cells transiently transfected with wild-type or mutated minigenes alone or in combination with 1.5× molar excess of U1snRNA variants or the engineered U7snRNA. Amplified products were separated on 2% agarose gel (M, 100 bp molecular weight marker). Transcripts were validated by sequencing, whose electropherograms are reported below. The green arrow indicates the c.386G > A mutation.

To create an experimental model useful to test correction strategies, we exploited the transient expression of mouse OTC minigenes (Figure 1A) in mouse hepatoma cells (Hepa1-6), chosen because OTC is physiologically expressed in the liver. As expected from previous minigene studies [23], the c.386G > A substitution remarkably decreased the proportion of correct transcripts and led to exon 4 skipping or the usage of a cryptic intronic 5′ss at position +49. 

To redirect the spliceosome to the defective 5′ss, we exploited an antisense strategy based on a modified U7snRNA, an approach successfully used by us in different gene contexts [25,26,27].

However, co-expression of the OTC^mut^ minigene with a U7snRNA variant designed to target and mask the cryptic 5′ss (U7^O^, Figure 1A) resulted in a reduction in the cryptic 5′ss usage but increased exon 4 skipping, with a negligible impact on correct exon 4, definition. A similar effect was also reported with the use of an antisense oligonucleotide [23]. This observation indicated that U7^O^ has been assembled into a functional snRNP, as shown by effects on cryptic 5′ss usage, but underlined an aberrant splicing mechanism due to impaired recognition of the mutated 5′ss, where the interaction with the U1snRNP is crucial. Therefore, we designed a panel of U1snRNA variants (Figure 1A) with increased complementarity with the authentic exon 4 5′ss (compensatory U1snRNA, U1^O^), or targeting an exonic (U1^E^) or downstream intronic sequences (ExSpeU1 U1^O1^, U1^O2^ U1^O3^). Notably, with the exception of the U1E, the co-expression of the compensatory or the exon specific U1snRNA variants with the OTC^mut^ minigene restored exon 4 inclusion and resulted in a splicing pattern comparable to that of the OTC^wt^ construct (Figure 1B). On the other hand, the delivery of an engineered U7snRNA designed to mask the same intronic sequence recognized by the U1^O1^ to U1^O3^ was ineffective. These data further support a mechanism in which the ExSpeU1s rescue proper exon definition by interacting with the spliceosome and not by simply interfering with a negative splicing regulatory element [19].

Altogether these data indicated that the c.386G > A mutation can be rescued by U1snRNA variants and led to the selection of the ExSpe U1^O3^ for studies in *spf*/*ash* mice since targeting a poorly conserved intronic sequence instead of the 5′ss sequence would most likely result in a higher gene specificity.

### 2.2. AAV8-Mediated Delivery of ExSpeU1^O3^ Partially Rescues OTC Expression

**Figure 2:**
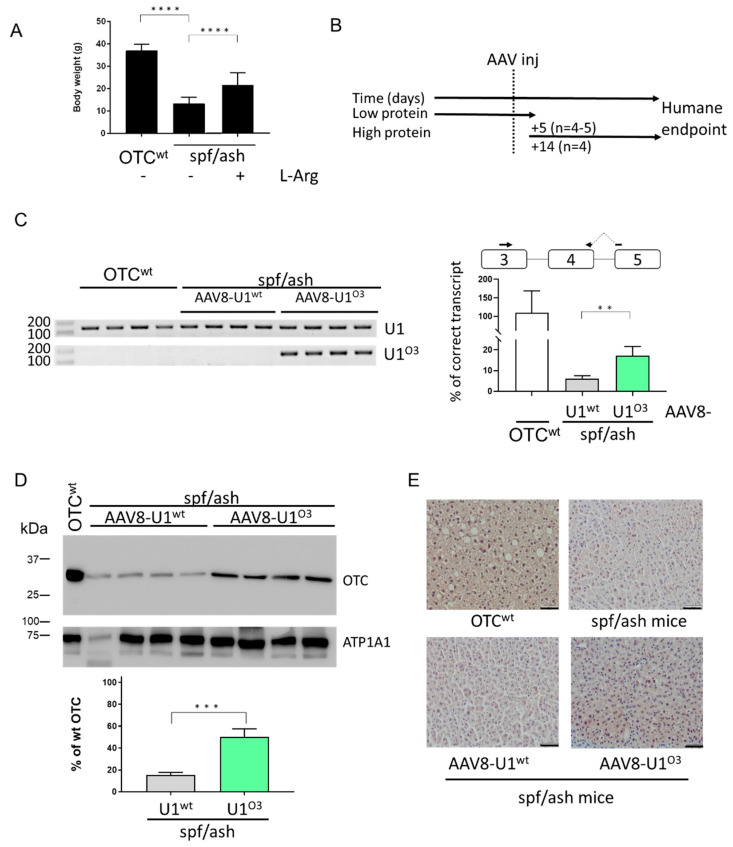
Exon Specific U1snRNA (ExSpeU1)snRNA U1^O3^ restores OTC expression and ameliorates the *spf*/*ash* mouse phenotype. Asterisks represent P values: ** *p* ≤ 0.01, *** *p* ≤ 0.001, **** *p* ≤ 0.0001 (**A**) Mean body weight of OTC wt and *spf*/*ash* mice kept on a low protein diet with or without the supplementation of sucrose and L-Arginine. (**B**) Schematic representation of the protocol designed to perform the experiments in mice and exploiting the adeno-associated virus (AAV8)-mediated delivery of the U1^O3^. Five or fourteen days post-injection of 1 × 10^13^ vg/kg body weight of AAV8-U1^O3^, mice were challenged with a high protein diet. (**C**) Evaluation of U1snRNA expression (left) and correctly spliced OTC transcripts (right) in mouse liver samples. The schematic representation of the *OTC* gene is reported together with the exploited primers (arrows). The relative amount of correctly spliced transcripts in mice injected with the AAV8-U1^O3^ as compared to those in wild-type mice (%) is reported as mean ± SD from three independent experiments. (**D**) Western blotting analysis in liver homogenates from *spf*/*ash* mice injected with the AAV-U1^wt^ or AAV-U1^O3^. Each line is an individual mouse. For each experimental group, four mice were randomly selected. The mouse ATPase Na+/K+ Transporting Subunit Alpha 1 (ATP1A1) was used as load control. The virtual protein marker, reporting the molecular size of bands, is reported on the left. The bars represent the relative amount of OTC protein as compared to that of the positive control (wild-type mouse liver), quantified by densitometric analysis. Results are reported as mean ± SD from three independent experiments. (**E**) Immunohistochemical analysis of OTC expression in mouse liver sections. Pictures represent examples of liver sections stained with a specific anti-OTC antibody (brown). Images are taken at 20× magnification. Scale bar, 50 µm.

Prompted by the encouraging results in vitro, the U1^O3^ efficacy was challenged in the *spf*/*ash* mice by monitoring the level of OTC expression in the liver. Adult *spf*/*ash* male mice were initially kept on a low protein diet to avoid the development of a severe OTCD phenotype due to toxic ammonia levels. At 8–10 weeks, mice weighed 13 ± 2.8 g, while wild-type (wt) littermates kept on the same diet, gained weight more rapidly, and weighed 37 ± 2.9 g, confirming the growth impairment [28]. To maintain a normal rate of protein synthesis, *spf*/*ash* male mice were kept on low protein chow but supplemented with sucrose and L-Arginine, which led to a partial normalization of body weight (21 ± 5.5 g) (Figure 2A). Male mice were injected with 1 × 10^13^ vector genomes/kg of adeno-associated virus (AAV8)-U1^wt^ (*n* = 9) or AAV8-U1^O3^ (*n* = 8) and challenged five or fourteen days later with a high protein diet (Figure 2B). Moreover, to provide evidence that the U1-mediated OTC rescue is mediated by the U1^O3^ binding and not by U1snRNP overexpression, the endogenous murine U1snRNA (U1^wt^) was exploited as control, also because both experimental groups shared the AAV transduction effects in hepatocytes, where the OTC expression is evaluated.

The transduction efficiency, as well as the expression of the U1snRNA and the associated OTC levels in the liver, was investigated in mice at the humane endpoint, which was reached slightly later in those treated with the AAV8-U1^O3^ (day eight) than those with the AAV8-U1^wt^ (day five). 

The transduction efficiency of the AAV8-U1 has been evaluated by two complementary approaches. In particular, the immune-histochemical analysis revealed that the Green Fluorescent Protein (GFP) staining, a marker of viral transduction, was comparable among mice irrespectively of the AAV8-U1 injected and not homogeneously distributed, with large variations in the number of GFP-positive hepatocytes across liver sections (Figure S1A), as previously observed by us [22,29] and others [30,31]. This finding was consistent with the AAV gene copy number that appeared to be comparable among experimental groups (Figure S1B). However, this analysis demonstrated a modest transduction efficiency (average 0.14 AAV copies/diploid genome) compared to that (>2) observed by others [32,33].

Despite the low transduction efficiency, the evaluation of correctly spliced OTC transcripts by a tailored qPCR approach revealed a significant increase in *spf*/*ash* mice injected with AAV8-U1^O3^ as compared to those observed in mice receiving the AAV8-U1^wt^ treatment (from 6.1 ± 1.4% to 17.2 ± 4.5% of those in wild-type mice, *p* = 0.0033) (Figure 2C, right panel). Consistently, Western blotting analysis in liver homogenates revealed a ~3-fold increase in OTC expression in *spf*/*ash* mice injected with AAV8-U1^O3^ compared with those injected with the AAV8-U1wt (from 15.1 ± 2.6% to 50.1 ± 7.4% of that of the wild-type mouse used as a positive control; *p* = 0.0001) (Figure 2D). To further strengthen the evidence for the U1^O3^-mediated rescue, we also performed OTC immunostaining in liver sections, which revealed a stronger OTC signal in AAV8-U1^O3^ treated mice as compared with those injected with the U1wt (Figure 2E).

As expected, the expression of the U1^O3^ through appropriately designed primers was clearly detectable only in *spf*/*ash* mice injected with AAV8-U1^O3^ (Figure 2C, left panel). Moreover, the qPCR and Western blotting confirmed that the c.386G > A (p.R129H) mutation is compatible with residual OTC expression levels, as previously reported for the *spf*/*ash* mouse model as well as in patients [23] and explaining a severe phenotype in high protein diet conditions. 

Taken together, these data indicated that the AAV8-mediated delivery of the ExSpeU1^O3^ in *spf*/*ash* mouse liver partially rescues OTC splicing and protein levels, while the limited transduction efficiency might explain the discrepancy between the efficiency of splicing correction in vitro versus in vivo. 

## 3. Material and Methods

### 3.1. Minigene Constructs and Splicing Assays

To create the pOTC^wt^ vector, the mouse *OTC* exon 4 and the flanking intronic sequences (from position −546 to +615) were amplified from genomic DNA of a *CL57BL6* mouse with primers 4F-4R and cloned into the expression vector pTB by using the *NdeI* restriction sites. The *OTC* c.386G > A mutation was inserted by mutagenesis (QuickChange II Site-Directed Mutagenesis Kit, Stratagene, La Jolla, CA, USA). Expression vectors for the U1snRNA and U7snRNA variants were created, as previously reported [12]. The U1 coding cassettes, either the endogenous U1 (U1^wt^) or the ExSpeU1 (U1^O3^), were cloned into an AAV8 plasmid carrying the GFP gene under the control of the PGK promoter, thus generating the AAV8-U1^wt^ and AAV8-U1^O3^ plasmids

Mouse hepatoma Hepa1-6 cells were cultured and transfected on 12-well plates [34] with 500 nanograms of each minigene construct alone or in combination with a molar excess (1.5×) of the pU1/pU7 plasmids. Total RNA was isolated 24 h post-transfection with Trizol (Life Technologies, Carlsbad, CA, USA), reverse-transcribed with RT-MLV (Life Technologies, Carlsbad, CA, USA) using random primers. cDNA was amplified using the plasmid-specific primers Alfa and Bra. All constructs and transcript amplicons were validated by direct sequencing. Sequences of oligonucleotides are provided in Table S1.

### 3.2. Procedures in Mice 

The AAV8-coding plasmid was created to harbors the coding cassettes for both the U1snRNA (wild type or engineered one) and GFP genes under the control of the natural or Phosphoglycerate kinase 1 (PGK) promoters, respectively. An adenovirus-free transient transfection method [35] was exploited to generate deno-associated virus serotype 8 (AAV8), and a vector genome titer was determined, as previously described [22].

The *spf*/*ash* mouse model was obtained from Jackson Laboratories (JAX stock #001811) and subsequently bred in the Animal Research Institute of the Academic Medical Center Amsterdam. The study design and animal care and handling were approved by the Institutional Animal Care and Use Committee of the Academic Medical Centre of the University of Amsterdam (permit AVD118002016775, study ALC249AB, approved 3 January 2018). Wild-type littermates were used as control. OTC deficient animals were maintained on a low protein diet and drinking water supplemented with arginine and sucrose. OTC deficient male mice, 8-16 weeks old, received a retro-orbital injection of 1 × 10^13^ vg /kg of AAV8-U1^O3^ or AAV8-U1^wt^. Five days (4 mice injected with AAV8-U1^O3^ and 5 mice with AAV8-U1^wt^) or two weeks (4 mice/group) after injection, mice were put on a high protein diet, and the effect on body weight and overall appearance was monitored over time. A body weight loss of 20% of their maximally obtained weight was considered the humane endpoint, and mice were sacrificed. At this stage, blood samples and organs were harvested. 

The study design and animal care and handling were approved by the Institutional Animal Care and Use Committee of the Academic Medical Centre of the University of Amsterdam.

### 3.3. Evaluation of OTC and U1^O3^ Expression in Mice

Total liver RNA was isolated and used to generate cDNA as described above using random primers.

Levels of correctly spliced OTC transcripts were determined by quantitative PCR (qPCR) with SsoAdvanced Universal SYBER Green Supermix (Bio-Rad, Hercules, CA, USA) on diluted cDNA (1:10) with primers mOTCex3-mOTCwtex4 (Supplementary Table S1) on a CFX connect qPCR system (Bio-Rad, Hercules, CA, USA). Each sample was run in duplicate. Cq and melting curves were acquired by the use of Bio-Rad CFX Manager 3.1 software (Bio-Rad, Hercules, CA, USA). The mRNA levels were expressed as the relative expression index of 2-DDCt. Values were expressed as mean fold change standard error of the mean. OTC expression in control mice (C57BL/6) was used as a reference.

The expression of U1^O3^ and U1^wt^ was evaluated on diluted cDNA (1:10) by a semi-quantitative PCR using primers U1O3Ex and U1Ex (Supplementary Table S1) followed by agarose gel electrophoresis.

OTC protein expression in mouse liver sections was detected by immunostaining of liver sections [22] using a rabbit anti-OTC polyclonal (1:200 dilution; Novus Biologicals, Centennial, CO, USA; #NBP1-87408) and a polyclonal anti-rabbit IgG-HRP as the secondary antibody (Immunologic, Duiven, The Netherlands; DPVB110 HRP) or by Western blotting (100 ug of liver homogenates) using 1:1000 dilution of the anti-OTC polyclonal antibody.

### 3.4. Determination of AAV Liver Transduction Levels by GFP Expression in Liver Slices and GFP Gene Copies per Liver Cell

The presence of the GFP coding gene, under the control of the PGK promoter, in the same AAV8 vector delivering the U1snRNA cassette, allowed the determination of AAV/GFP gene copies per cell by using qPCR, as previously reported [22]. Briefly, the GFP gene copy number in genomic liver DNA was determined using two GFP-specific primers (Supplementary Table S1), using a standard curve created by spiking linearized AAV8-U1^wt^ plasmid in mouse genomic DNA isolated from the liver of a non-treated mouse. The AAV8 vector copy number was calculated using the number of double-stranded DNA of diploid genomes normalized for the amount of genomic DNA. The lower limit of detection was 18 GFP gene copies per 10^4^ genomes. GFP staining was performed as previously described [22].

## 4. Conclusions

Through investigations in a splicing-defective mouse model of ornithine transcarbamylase deficiency, we provided the early proof-of-principle that an exon specific U1snRNA variant (U1^O3^) can be exploited in vivo to rescue OTC expression impaired by a splicing defect. It is worth noting that this partial recovery was obtained in the presence of a modest transduction efficiency of hepatocytes, which may lead to an underestimation of the potential of the AAV8-U1^O3^ treatment. These data lay the foundation for further in vivo studies aimed at evaluating the therapeutic potential of ExSpeU1 for OTCD forms caused by aberrant splicing.

## 5. Patents

M.P. is the inventor of a patent (PCT/IB2011/054573) on modified U1snRNAs.

## Supplementary Materials

The following are available online at https://www.mdpi.com/1422-0067/21/22/8735/s1.
