# Supplementary material for: An Exon-Specific Small Nuclear U1 RNA (ExSpeU1) Improves Hepatic OTC Expression in a Splicing-Defective spf/ash Mouse Model of Ornithine Transcarbamylase Deficiency
Source: Int J Mol Sci. 2020 Nov 19;21(22):8735. doi: 10.3390/ijms21228735 (PMC7699343; doi:10.3390/ijms21228735)
Supplement: Supplementary file 1 [file ijms-21-08735-s001.pdf]

International Journal of *Molecular Sciences*

MDPI

*Supplementary*

# An Exon-Specific Small Nuclear U1 RNA (ExSpeU1) Improves Hepatic OTC Expression in a Splicing-Defective *spf/ash* Mouse Model of Ornithine Transcarbamylase Deficiency

**Dario Balestra [1,*], Mattia Ferrarese [1], Silvia Lombardi [1], Nicole Ziliotto [1,4], Alessio Branchini [1], Naomi Petersen [2], Piter Bosma [2,3], Mirko Pinotti [1] and Stan F.J. van de Graaf [2,3]**

1 Department of Life Sciences and Biotechnology and LTTA, University of Ferrara, 44121 Ferrara, Italy; frrmtt1@unife.it (M.F.); lmbslv@unife.it (S.L.); zltncl1@unife.it (N.Z.); brnlss@unife.it (A.B.); pnm@unife.it (M.P.)

2 Tytgat Institute for Liver and Intestinal Research and Department of Gastroenterology and Hepatology, Amsterdam UMC, University of Amsterdam, 1105 AZ Amsterdam, The Netherlands; n.petersen@amc.uva.nl (N.P.); p.j.bosma@amsterdamumc.nl (P.B.); k.f.vandegraaf@amsterdamumc.nl (S.F.J.v.d.G.)

3 Amsterdam Gastroenterology Endocrinology Metabolism, Amsterdam UMC, 1105 AZ Amsterdam, The Netherlands

4 School of Medicine and Surgery, University of Milano-Bicocca, 20900 Milan, Italy; nicole.ziliotto@unimib.it

* Correspondence: blsdra@unife.it; Tel.: +39-0532-974485

Received: 23 September 2020; Accepted: 17 November 2020; Published: 19 November 2020

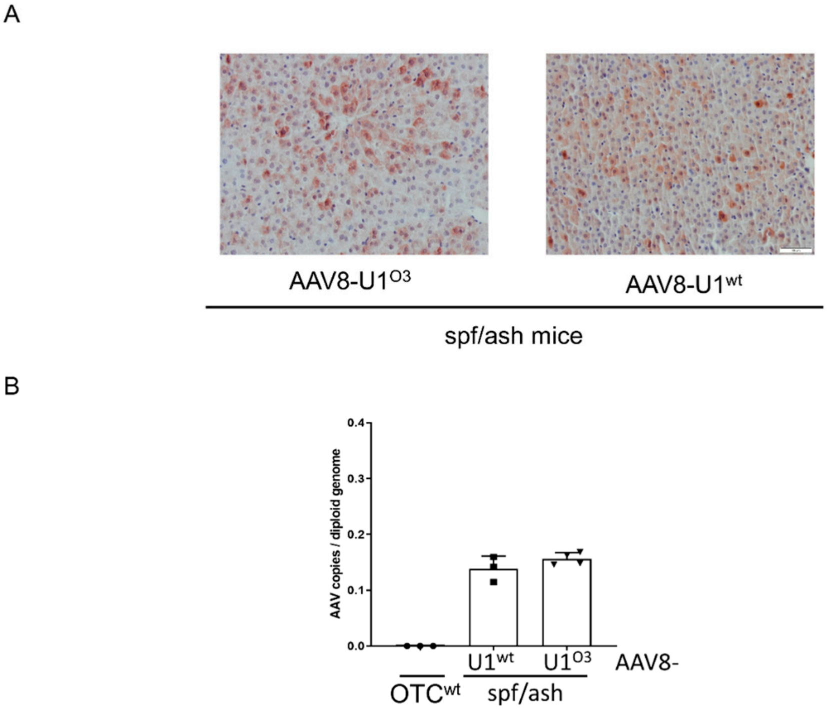


**Supplementary Figure S1. (A)** eGFP expression in livers of spf/ash mice injected with the AAV8-U1$^{wt}$ or AAV8-U1$^{O3}$. Representative examples of liver sections for both animal models are shown. Images are taken at 20× magnification. Scale bar, 50 µm. **(B)** eGFP gene copy number in liver expressed as AAV copies per diploid mouse genome.

**Supplementary Table S1.** Sequences of oligonucleotides.

| **Creation of mouse OTC minigene** | | |
|---|---|---|
| **4F** | forward | tttaaacatatgctgtgtaacctggaagctctatgat |
| **4R** | reverse | tttaaacatatgccttcaacattgtccttgttatttc |
| Mutant mouse OTC minigene | | |
| **OTC mut F** | forward | ctcacagacaccgctc**a**gtttgtaaaacttttc |
| **OTC mut R** | reverse | gaaaagttttacaaac**t**gagcggtgtctgtgag |
| Engineered U1snRNA variants for mouse OTC 5′ss | | |
| **$U1^{O}$ F** | forward | aggcccaagatctgatACAAACCGAgcaggggagataccat |
| **$U1^{O1}$ F** | forward | aggcccaagatctgatTTTACAAACgcaggggagataccat |
| **$U1^{O2}$ F** | forward | aggcccaagatctgatAAGTTTTACgcaggggagataccatgatca |
| **$U1^{O3}$ F** | forward | aggcccaagatctgatAGAAAAGTTgcaggggagataccatgatca |
| Engineered U7snRNA variant for mouse OTC 5′ss | | |
| **$U7^{O}$** | forward | acagaggcctttccgcacttttaaactaacccatcagagaatttttggag |
| Plasmid-specific primers used to analyze splicing pattern | | |
| **alfa** | forward | caacttcaagctcctaagccactgc |
| **bra** | reverse | taggatccggtcaccaggaagttggttaaatca |
| Primers used to assess in vivo splicing | | |
| **mOTCex3** | forward | aagaagtactcgaacaagactgtcc |
| Primers used to evaluate the correctly spliced transcripts | | |
| **mOTCwtex4** | reverse | catctgtcatgctagataagacacgag |
| Primers used to evaluate the titer of AAV vectors | | |
| **eGFP** | forward | GCGGGGCAAGTGACCGTGTG |
| | reverse | TGCGCAAACCCAGGGCTGCC |
| Primers used to evaluate U1 expression | | |
| **$U1^{wt}Ex$** | forward | agatctcatacttacctg |
| **$U1^{O3}Ex$** | forward | agatctgatagaaaagtt |
| **U1Ex** | reverse | gaacgcagtcccccactaccac |
| Primers used to evaluate the AAV8 gene copy number | | |
| **GFP** | forward | catggtcctgctggagttc |
| | reverse | gtcaggtcagcttggtcttc |

The NdeI restriction site is underlined; mutated nucleotides are in bold; the replaced 9 nucleotides of 5′ tail of U1snRNA variants are indicated in upper case.
